# Innovative Alveolar Ridge Preservation Surgical Technique with Immediate Dental Implant Placement: A Retrospective Case Report of 1-Year Follow-Up

**DOI:** 10.1055/s-0043-1772676

**Published:** 2023-11-23

**Authors:** Andrea Grassi, Daniele Monica, Elio Minetti, Andrea Ballini, Francesco Gianfreda, Patrizio Bollero, Marco Cicciù, Filiberto Mastrangelo

**Affiliations:** 1 Private Practice Dental Clinic, Reggio Emilia, Italy; 2 Department of Clinical and Experimental Medicine, Dental School, University of Foggia, Foggia, Italy; 3 Private Practice and Professor a c. University of Milan, Milan, Italy; 4 Department of Industrial Engineering, University of Rome “Tor Vergata”, Rome, Italy; 5 Department of System Medicine, Dental School, University of Rome “Tor Vergata”, Rome, Italy; 6 Department of General Surgery and Surgical-Medical Specialties, Dental School, University of Catania, Catania, Italy

**Keywords:** alveolar ridge preservation, periosteal inhibition, modified periosteal inhibition, cortical lamina, dental implant, implant survival

## Abstract

Following tooth extraction, the alveolar ridge undergoes morphological and dimensional changes, including a clot formation that is gradually replaced by granulation tissue. Studies indicate that both horizontal and vertical ridge dimensions decrease after extraction; however, these changes can be mitigated through grafting with biomaterials and barrier membranes. Alveolar ridge preservation (ARP) techniques are employed to counteract bone resorption postextraction, encompassing periosteal inhibition and modified periosteal inhibition (MPI) techniques. The Degidi clot chamber technique offers a means to achieve biomaterial-free extraction sockets, promoting healing and osteointegration. This study aims to present the first rehabilitation of a postextraction dental implant in the maxilla using an innovative ARP procedure via a MPI technique. The technique does not involve autologous or heterologous grafting materials; instead, a cortical lamina and a customized screw are used in conjunction with the blood clot. The primary objective is to protect the vestibular cortical bone from preosteoclastic aggression, which can trigger bone resorption. The technique employs a 0.5-mm cortical lamina to mechanically shield the vestibular cortical bone, preventing vestibular cortical bone resorption and increasing its thickness without the need for biomaterial insertion, relying on the blood clot. The effectiveness of the technique was assessed through a 12-month postimplantation cone-beam computed tomography scan, revealing a 0.5-mm increase. Although based on a single case, the 1-year follow-up results are promising, and further studies are warranted to validate the technique's efficacy.

## Introduction

The rehabilitation of edentulous sites through the use of implants is widely used. The studies of Gupta et al showed a high success rate, around 97% after a follow-up of 10 years. 1 Gay and colleagues through a retrospective study evidenced the survival rate of implants placed in the native bone and regenerated bone after 5 and 10 years were 92 and 87% for implants placed in the native bone, and 90 and 79% for implants placed in the grafted bone. 2 After tooth extraction, on the edentulous site occur morphological and dimensional changes of the alveolar ridge due to qualitative and quantitative modification. 3 4 5 In physiological conditions a clot is formed, consisting of blood cells, serum, and saliva. The blood vessels are closed by the thrombus and a network of fibrin forms, within the first day, constitutes the scaffold on which neutrophil, monocyte, and fibroblast granulocytes begin to migrate. In the central area, lymphocytes and leukocytes begin the hemolysis process, through which the clot is slowly replaced by granulation tissue. 6 7 8 Several studies have reported a reduction in the horizontal ridge size in postextractive sockets from 2.6 to 4.6 mm 4 9 10 ; while the average vertical ridge height reduction was about 1.24 mm. 4 11 Grafting alveolar socket with biomaterials and the use of barrier membranes can reduce the degree of dimensional alterations. 12 13 In alveolar socket preservation (ASP) procedure, autogenous bone is widely accepted as the gold-standard bone grafting material because it owns the characteristics of osteoconduction, osteoinduction, and osteogenesis. 14 15 Moreover, heterologous bone applied to ASP practice is employed, such as deproteinized bovine bone in association with human fibrin and collagen glue. 4 Due to the Tooth Transformer device the patient's extracted tooth is transformed into autologous biomaterial. The device reduces the crystallinity of hydroxyapatite and obtains the morphogenetic proteins and growth factors present in dentine. The resulting particulate determines the mechanism of osteoinduction. 16 Alveolar ridge preservation (ARP) techniques are used to counteract the physiological bone ridge resorption that occurs after tooth extraction. The periosteal inhibition preserve the thickness with the use of a dense-polytetrafluoroethylene (d-PTFE) membrane placed between the vestibular periosteum and the cortical bone, and no bone grafts inserted in the sockets. A new technique used with this purpose is the modified periosteal inhibition (MPI). This MPI technique aims to preserve the vestibular bone by exploiting periosteal inhibition and to increase its thickness by bonding a single or double layer of OsteoBiol Lamina Soft (Tecnoss, Giaveno, Italy) 0.5 mm thick. The average increase obtained with this procedure, experienced in nine sockets, was 0.41 ± 0.21 mm. The initial buccal bone thickness was 1.18 ± 0.57 mm. The preoperative ridge width was 10.74 ± 1.54 mm, while the postoperative width was 11.16 ± 1.57 mm. 17 The focus of the following case report is the absence of use of any biomaterial in the socket after the extraction. The technique is based on the concept of Degidi's clot chamber, which is a surgical approach used in dental implantology to increase the success rate of dental implants. The technique involves leaving intact the blood clot formed in the cavity created by tooth extraction, instead of removing it and suturing the surgical wound. The blood clot acts as a bridge between the surrounding bone and the dental implant, facilitating healing and promoting the formation of new bone tissue around the implant with the purpose to guarantee the osseointegration. 18 19 20

The aim of the present study was to describe the first postextraction dental implant rehabilitation in maxillary site treated with an innovative ARP procedure through a MPI technique without insertion of autologous or heterologous graft materials and customized screws.

## Case Report

### Study Design

The following case study report was performed in a private clinic (Dott. A. G.) in compliance with the patient, who satisfied the inclusion criteria, who was included in the present study: male, 62 years old, nonsmoker, and in general good health. Written informed consent was signed by the patient for the clinical procedure and for the present study. The patient was provided with a prophylaxis starting the day before the surgery for 6 days, with 2 g of amoxicillin and clavulanic acid. Preoperative cone-beam computed tomography (CBCT) was performed.

*Inclusion criteria* :

- Age > 18 years old
- General good health (American Society of Anesthesiologists I–II)
- Adequate oral hygiene (full mouth plaque score ≤ 20%, full mouth bleeding score ≤ 20%)
- Presence of one or more hopeless teeth requiring extraction

*Exclusion criteria* :

- Pregnancy or lactating period
- Untreated periodontitis
- Osteometabolic disease
- Intravenous bisphosphonates therapy
- Chemotherapy or radiation therapy history of the neck–head area
- Heavy smokers (> 15 cigarettes/per day)
- Absence of buccal bone plate

### Surgical Protocol

**Fig. 1:**
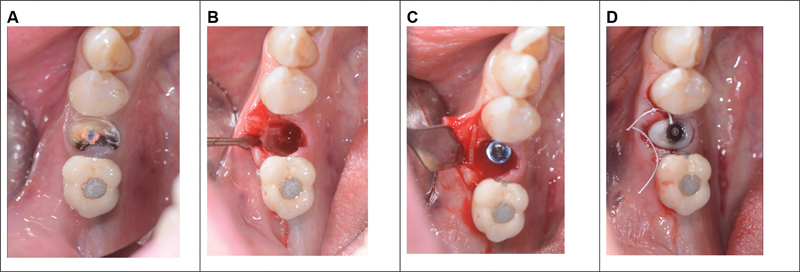
( **A** ) Preoperative sites. ( **B** ) Alveolar socket. ( **C** ) Implant placement with the cortical lamina. ( **D** ) Custom screw.

Following local anesthesia utilizing a 4% articaine solution containing 1:200,000 adrenaline, an atraumatic extraction was executed with preservation of the buccal bone, and the alveolar socket was meticulously debrided. Subsequently, after performing incisions on the papillae, an intrasulcular incision was made on the vestibular aspect of the extraction socket, extending to the mesial and distal midtooth region using a #15c surgical scalpel. A full-thickness flap was elevated and the periosteum was disconnected with the aid of microcollars. Once debridement was performed, clot formation was allowed, following the criteria of the clot chamber described by Degidi, in order to avoid the insertion of any biomaterial. The clot was left in place and after achieving stability, a single customized screw-type abutment T3, 3i (Zimmer Biomet) implant with a diameter of 4.1 mm was inserted, and screwed in place. The implant was oriented in palatal position and was temporarily cemented, following a delayed loading. A cortical lamina, OsteoBiol Lamina Soft 25 × 25 mm, was placed between the periosteum and cortical bone to prevent both vertical and horizontal resorption. The cortical lamina was meticulously placed and, if necessary, contoured until a perfect fit was achieved. If the bony peaks were higher than the level of the buccal bone plate, it was possible to obtain a shape that partially lodged under the gingival papillae. The edges were rounded to minimize the risk of soft tissue trauma during the healing phase. The thrombin component of human fibrin glue (Tisseel, Baxter Healthcare Corporation, Deerfield, Illinois, United States) was diluted with 9 mL of sterile saline, reducing the international thrombin units from 500 to 50. Two to three drops of diluted glue were placed on the cortical lamina, which was then locked in place via simple pressure to the vestibular bone, aligning it with the extraction socket border. The papillae were sutured with two sling stitches. The sutures were removed after 7 to 8 days (Fig. 1).

### Radiographic Evaluation

CBCT exams were performed before dental extraction (baseline) and after 1 year with Carestream 8100 3D (Carestream Dental, Atlanta, Georgia, United States). The measurements were carried out by a single-blinded operator (A.G.). Taking the remaining teeth as a reference, we positioned ourselves in the same coronal cuts, measuring the bone thickness.

#### Case Report

**Fig. 2:**
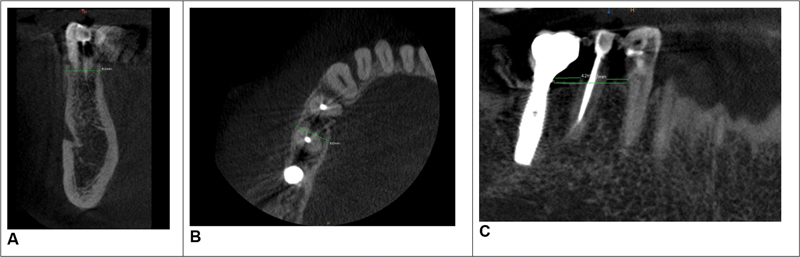
( **A** – **C** ) Cone-beam computed tomography (CBCT) images of patient 1, before the surgery.

The preoperative ridge width was 8.00 mm (Fig. 2). The initial interproximal bone distance of the zone 34 and 35 was 4.2 mm and in the zone of 35 and 36 was 3.7 mm. The total distance of the affected area was 12 mm. No volume contraction of the healing sockets was measured.

## Results

### Radiographic Linear Measurements

**Fig. 3:**
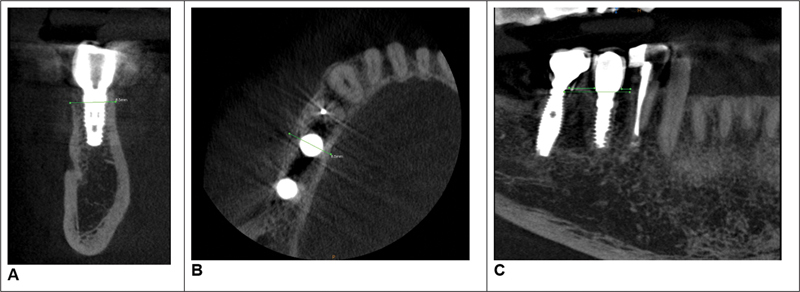
Cone-beam computed tomography (CBCT) images of patient 1, after 1 year.

After 1 year from the placement of implant, the postoperative width was 8.5 mm, an improvement of +0.5 mm (Fig. 3). The interproximal distance of the zone 34 and 35 was 1.7 mm and zone 35 and 36 was 4.7 mm. The total distance of the affected area was 12 mm.

### Clinical Outcomes Follow-Up

**Fig. 4:**
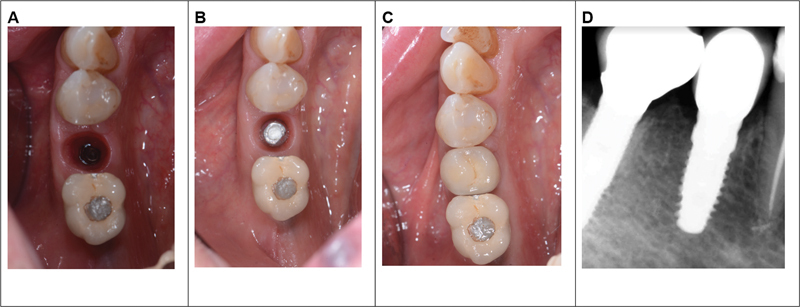
( **A** and **B** ) Postoperative results. ( **C** ) Implant loading. ( **D** ) Radiological evaluation 1 year after loading.

After 6 months, there is a transition from a customized screw-type abutment to a curved max abutment with temporary cementation. It is important to note that by modifying the supraimplant geometries, reducing the diameter of the abutment, and moving the prosthetic margins away from the implant, soft tissues benefit significantly (Fig. 4).

## Discussion

According to a study conducted by Trombelli et al in 2018, the loss of alveolar bone after tooth extraction can vary from 29 to 63% in the first 6 months, with an average of 40%. However, this process can continue for several years, resulting in further reduction of bone volume. 21 Numerous studies have reported a reduction in horizontal ridge dimension in postextraction sockets from 2.6 to 4.6 mm, 4 9 10 while the average reduction in vertical ridge height was approximately 1.24 mm. 4 11 Several techniques are used to overcome bone loss, such as ASP, in which the use of biomaterials or autologous bone has resulted in a decrease in the amount of resorbed bone. This technique allows limiting the amount of bone that is resorbed after an extraction. ASP is a procedure used in dentistry to preserve the integrity of the alveolus after tooth extraction. This procedure aims to preserve the quantity and quality of alveolar bone around the former site of dental extraction, avoiding bone resorption and promoting subsequent prosthetic or implant rehabilitation. ASP has become a common practice in dentistry in recent decades, thanks to its effectiveness in helping prevent bone loss and maintaining smile aesthetics. Patients undergoing ASP have a higher chance of long-term success in prosthetic or implant rehabilitation, as the preservation of alveolar bone reduces the need for bone reconstruction interventions. There are several techniques to perform the ASP procedure, including the use of alveolar filling materials, bone regeneration membranes, and bone growth factors. Alveolar filling material, such as bone substitutes or fibrin clots, contributes to maintaining the integrity of the alveolus and reducing the risk of bone resorption. Bone regeneration membranes can also help preserve the integrity of the alveolus and promote bone growth, as well as the use of bone growth factors such as bone morphogenetic protein (BMP)-2, which are useful for promoting bone regeneration after ASP. 8 Guided bone regeneration (GBR) is a surgical technique that involves the use of membranes in association with filling materials or not, to recreate adequate regeneration of missing bone tissue. 22 GBR involves the use of membranes or filling materials to guide bone regeneration. This technique can be used for the treatment of bone defects caused by periodontal diseases, fractures, tooth extractions, or for the preparation of the dental implant site. GBR can be performed using collagen membranes, resorbable or nonresorbable membranes, filling materials such as hydroxyapatite, tricalcium phosphate, or deproteinized bovine bone mineral, and growth factors such as BMP-2. 8 ARP is a procedure to halt or minimize alveolar ridge resorption after tooth extraction for future prosthetic treatment, including dental implant placement. 23 ARP, instead, involves the combination of surgical techniques and the use of filling materials for periodontal tissue regeneration. 22 This technique can be used for the treatment of periodontal defects caused by periodontal diseases, trauma, or for preparing the site for dental implantation. ARP can be performed using collagen membranes, regeneration matrices, growth factors such as platelet-derived growth factor, and the use of stem cells. 24 The use of biomaterials in GBR can lead to a significant increase in bone volume compared to natural healing, especially in cases of severe bone loss. 25 The biomaterials used can be of different types, such as synthetic bone substitutes, natural bone substitutes, or composites of these materials. According to the study conducted by Avila-Ortiz et al, 26 which aimed to evaluate the effectiveness of the surgical approach with ARP compared to the process of spontaneous healing of the socket. All results regarding the size of the dense bone were favorable to the use of ARP. These differences were statistically significant in terms of bone buccolingual width, midbuccal height loss, and bone ridge volumetric reduction. Volumetric analysis from the 14-week baseline showed almost double bone reduction in the control group (15.83% ± 4.48%) compared to the experimental group (8.36% ± 3.81%). Analysis of soft tissue volumetric variation in a subset of sites showed a similar reduction for both groups (control, 21.1% ± 8.83%; experimental, 19.61% ± 7.31%). 26

The authors called the present surgical technique “periosteal inhibition.” This surgical technique was used to preserve and increase the thickness of the vestibular bone after tooth extraction, in order to counteract the physiological resorption of the bone crest. The technique involves the use of a membrane placed between the vestibular periosteum and the cortical bone, and the insertion of a 0.5-mm thick cortical plate. The cortical plate is shaped and fixed with human fibrin glue, and a collagen sponge is inserted to stabilize the clot. CBCT exams are performed before dental extraction and after 4 months to evaluate the effectiveness of the technique. The technique achieved an average increase in vestibular bone thickness of 0.41 to 0.21 mm, and no early or late complications were observed. 17

The periosteal inhibition technique presented by the authors since 2019, both for alveolar preservation and immediate implant placement, shifts the focus from what happens inside the alveolus to what happens outside the bony cortices. The goal of this technique is no longer to fill the space created by tooth extraction, but to mechanically protect the vestibular cortical bone from preosteoclastic aggression. Preosteoclasts, which are attracted by the inflammatory-reparative situation, attach to the cortical bone and form osteoclasts, which begin to resorb it. 27 The MPI technique uses a 0.5-mm cortical lamina instead of a PTFE membrane that was suggested by some authors. 28 The advantages of this solution are many. The lamina can be left in place, eliminating the need to remove the PTFE after 4 months. Additionally, horizontal bone gain can be achieved, where normally hard tissue resorption was observed. CBCT performed 12 months after implant placement showed a 0.5-mm increase in the thickness of the alveolar crest, achieved without filling the perimplant gap, where the 0.9-mm vestibular cortical bone was at high risk of resorption. In the analyzed case report, it is noted how the 1-year follow-up reported an increase in bone cortical thickness of 0.5 mm, thus bringing the thickness from 8.00 to 8.5 mm, determining the success of the intervention after 1 year.

The use of cortical laminas in periodontal regeneration has shown promising results, as indicated by the following references. Festa et al 29 conducted a randomized controlled clinical study to compare the preservation of alveolar ridge dimensions following tooth extraction using porcine-derived xenograft (OsteoBiol GenOs) combined with OsteoBiol Lamina Soft versus extraction sites alone. After 6 months, the extraction sites exhibited greater buccolingual dimension reabsorption compared to the grafted sites, and the mean reduction in vertical ridge height in the control sockets was significantly higher than that in the grafted sites. The authors concluded that the combination of OsteoBiol Lamina Soft and xenograft was effective in minimizing the effects of extraction.

Several authors 30 31 have proposed the use of OsteoBiol Lamina instead of autogenous bone plates. OsteoBiol Lamina (Tecnoss) is a collagenated porcine cortical bone membrane. In a recent research, 32 the scanning electron microscope demonstrated its similarity to a semipermeable membrane due to the presence of holes and channels on the surface that favor revascularization of the grafted area, from both the flap side and the grafted site. It is worth noting that the role of the cortical lamina is similar to that of the bone used in the Khoury technique, but it is much thinner and more adaptable. In the Khoury technique, it is the cortical lamina that prevents periosteal proliferation in the deeper areas of the regenerated site, eliminating the need for collagen barriers. 33

This new technique would seem very interesting both for its microinvasiveness and for the possibility of speeding up bone healing processes. In fact, the insertion of a slow resorption graft could require a longer time for the socket to heal. 8 Future studies could expand the sample of patients taken into consideration or test the technique in addition to titrated implants, 34 different implant morphologies associated with different prosthetic connections, 35 36 and the association with osteocompaction techniques such as those used in crestal sinus lifts. 37

## Conclusion

This first case of immediate implant placement (MPI) has yielded a certainly encouraging result from both hard and soft tissue perspectives, preventing resorption of the vestibular cortical and even increasing its thickness. The thin and festooned soft tissues appear to be increased and healthy, without performing a connective tissue graft. Although limited to a single case with the use of a new technique, the results obtained after 1 year of follow-up are particularly promising with respect to both hard and soft tissues. Of course, many more cases and more complex studies will be needed to determine whether this technique can become a common practice in immediate implant placement or in patients with systemic deseases. 38 39
